# Chewing Patterns and Muscular Activation in Deep Bite Malocclusion

**DOI:** 10.3390/jcm11061702

**Published:** 2022-03-19

**Authors:** Maria Grazia Piancino, Alessandro Tortarolo, Laura Di Benedetto, Vito Crincoli, Deborah Falla

**Affiliations:** 1 Department of Surgical Sciences, C.I.R. Dental School, University of Turin, 10126 Turin, Italy; alessandro.tortarolo@unito.it (A.T.); laura.dibenedetto@unito.it (L.D.B.); 2 Department of Basic Medical Sciences, Neuroscience and Sense Organs, University of Bari, 70126 Bari, Italy; vito.crincoli@uniba.it; 3 Centre of Precision Rehabilitation for Spinal Pain (CPR Spine), School of Sport, Exercise and Rehabilitation Sciences, University of Birmingham, Birmingham B4 7DA, UK; d.falla@bham.ac.uk

**Keywords:** surface EMG, chewing pattern, jaw muscles, deep bite, Function Generating Bite

## Abstract

Background: Deep bite, a frequent malocclusion with a high relapse rate, is associated with craniofacial features that need to be considered in the course of orthodontic treatment. Methods: This study included 81 patients with deep bite malocclusion (11.4 ± 1.1 [yr.mo]; M = 32 and F = 49), and 14 age- and gender-matched controls (9.11 ± 1 [yr.mo]; M = 5 and F = 9). The patients with deep bite malocclusion were treated with functional therapy. The chewing cycles and masticatory muscle EMG activity were recorded concomitantly before treatment in both groups (*n* = 95). Following correction of the malocclusion, a second recording took place (*n* = 25). Results: The kinematic variables showed the same dependency on bolus hardness in those with deep bite and in the controls. The masticatory muscle EMG activity was increased in those with deep bite, but decreased as a result of functional treatment. The chewing patterns showed a tendency towards a reduced lateral component, which significantly increased after treatment, indicating that functional therapy impacts the neuromuscular coordination of mastication, as well as dental positioning. Conclusions: Deep bite is a complex malocclusion, involving alterations in chewing and masticatory muscle activity. Orthognathodontic treatment should not only consider and correct the teeth position, but should also address muscular hyperactivity.

## 1. Introduction

**Figure 1:**
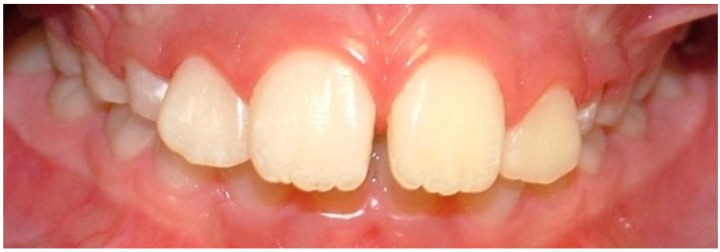
Deep bite in a mixed dentition patient.

Overbite (Figure 1) represents an important aspect of occlusion that has been the focus of therapeutic modifications since the early days of orthodontics [1]. The correction of overbite has also been used as an outcome to measure the quality of orthodontic treatment [2].

Deep bite is defined as a malocclusion in which the vertical overlapping of the anterior teeth is increased in comparison to the ideal value, often associated with decreased vertical facial dimensions [3], and is generally diagnosed in the presence of more than 3 mm of overbite. Deep bite is one of the most common malocclusions, with a reported prevalence of 46.2% in Germany [4], 41% in Italy [5,6,7], 21.6% in Colombia [8], and 24% in the United States [9]. Deep bite is notoriously prone to relapse after initially successful treatment [10,11].

Deep bite has different etiologies [12], and may be classified as skeletal, dentoalveolar, or dental. Skeletal deep bite is associated with hypodivergent craniofacial structures and an upper growth pattern of the mandibular condyle [13]; it is usually familial and exhibits characteristic dental traits, such as incisor supraocclusion, molar infraocclusion, and increased overjet [14,15,16]. The relationship between craniofacial morphology and masticatory muscle orientation, the presence of vertical malocclusion, and development has been extensively discussed in the literature. Indeed, the percentage of occupancy of different fiber types significantly differed in masseter muscle samples obtained from individuals with open bite and deep bite [17]. Interestingly, the isometric strength of the cervical flexor muscles, implicated in head posture, was influenced by variations in the vertical dimension [18]. Nonetheless, it is not clear whether strong masticatory muscles directly influence facial structure, or, rather, a genetically determined pattern of craniofacial growth informs the strength of the masticatory apparatus at the end of development [19]. Understanding the etiology of deep bite and its functional implications is crucial for obtaining stable results, after successfully treating the malocclusion. Craniofacial morphology, masticatory muscle orientation, and masticatory function are important considerations in both correct treatment planning and preventing relapse after successful treatment of deep bite cases. Dentoalveolar deep bite may be associated with lower lip hyperactivity, leading to lower anterior teeth overeruption and retroinclination [20,21,22].

**Figure 2:**
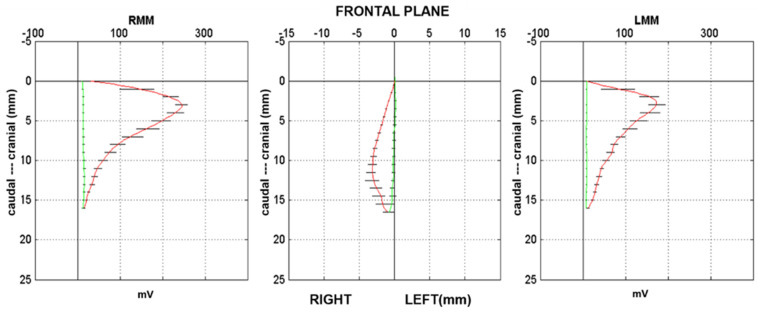
The masticatory kinematic pattern in the frontal plane (central plot), and the EMG envelope of the right and left masseter, plotted versus the vertical jaw displacement of a patient with deep bite malocclusion, during chewing of a hard bolus on the right side. The solid line, green for the opening pattern and red for the closing pattern, represents the average chewing cycle of 3 trials, lasting 10 s each; the black lines represent the standard deviation over the average cycle. Top: image of the deep bite malocclusion of one of the patients. Note the very high activity and the narrow pattern with very low standard deviation, especially in the occlusal phase of the closing pattern, indicating that the dental malocclusion and the hyperactivation of the masseter muscles restrict the mandibular movements. This is a functional malocclusion; the teeth position is the consequence of the cranial and muscular structure. mV, millivolt; RMM, right masseter muscle; LMM, left masseter muscle.

Previous investigations into the masticatory function of deep bite patients have shown alterations in chewing patterns. An early study reported that the vertical component of the chewing cycle was reduced after treatment [23]. More recently, the chewing cycle kinematics of young adults with deep bite were found to display lesser inferior and greater posterior excursions during opening, as well as slower jaw closing velocities, than those with normal occlusion [24]. The electromyography (EMG) amplitude of masticatory muscles was reported to be increased during chewing in people with deep bite [25,26] (Figure 2), and decreased after functional treatment [26]. This is consistent with different reports of greater masticatory muscle activation and increased masticatory efficiency in brachyfacial subjects [27,28,29]. In a previous article, we reported chewing cycle alterations and increased EMG activity of masticatory muscles in a deep bite patient with mixed dentition [30], as well as important changes after treatment, with the functional appliance Function Generating Bite (FGB).

It is, therefore, important to describe the alterations in chewing patterns and muscular activation together, following a repeatable and well-established protocol [31,32]. The aim of this work is to compare the chewing cycles and masticatory muscle activation of deep bite patients to those of control subjects, and to study the changes in the masticatory function associated with the treatment of deep bite with FGB, in order to test the hypothesis that deep bite is associated with chewing kinematics and alterations in masticatory muscle EMG activity.

## 2. Materials and Methods

This study included patients with deep bite malocclusion and subjects without malocclusion, who were referred to the orthodontic department of the University of Turin, Italy.

The inclusion criteria for the malocclusion group were the following: deep bite malocclusion (overbite >4 mm); mixed dentition. The exclusion criteria were the following: previous orthodontic treatment; signs or symptoms of dental or myofascial pain; signs or symptoms of cranio-mandibular disorders; prosthodontic rehabilitation of any kind.

Individuals included in the control group had mixed dentition and no malocclusion.

Patients with deep bite malocclusion were treated with functional therapy, i.e., FGB, individually manufactured and made of acrylic resin and resilient stainless steel, with anterior and posterior metallic bite planes, preventing the teeth from intercuspal contact. This appliance is fully removable and non-cariogenic, as it has an exclusively muscular anchorage, which allows it to exert alternating and self-regulating forces that correct both the dental malocclusion and the functional imbalance [32,33]. The appliance was worn day and night until deep bite correction, which was diagnosed on study casts. The malocclusion was considered corrected when the overbite reached 2 mm.

Patients without malocclusion did not receive any treatment.

The mean treatment time for the deep bite malocclusion group was 0.8 ± 0.2 [yr.mo]. The malocclusion was successfully corrected in all patients who underwent treatment. Chewing cycles were recorded before treatment in both groups (*n* = 95). Among the deep bite patients, 25 accepted and received functional therapy with FGB, and, following correction of the malocclusion, after a 6-month retention period, during which the appliance was worn at night, all 25 patients were available for a second recording of chewing cycles (*n* = 25).

### 2.1. Registration of the Masticatory Function

The masticatory function was registered with a kinesiograph (K7-I, Myotronics Inc. Tukwila, WA, USA), which measures jaw movements with an accuracy of 0.1 mm. This device includes lightweight (4 oz.) head-worn gear, with multiple sensors that track the motion of a tiny magnet attached to the lower inter-incisal point, by means of food-safe sticky wax. The K7-I was interfaced with a computer for data storage and subsequent analysis.

### 2.2. Electromyography

Surface EMG signals were recorded from the masseter muscles of both sides, with a multi-channel EMG amplifier (Myotronics Research Inc., Tukwila, WA-USA; bandwidth 45–430 Hz per channel). This EMG amplifier is part of the K7-I WIN Diagnostic System. The relatively large high-pass frequency in EMG recordings was selected to reduce low-frequency movement artifacts during chewing. Two electrodes (Duotrode silver/silver chloride EMG electrodes, Myotronics) were located on the masseter and temporalis anterior muscles of both sides, with an interelectrode distance of 20 mm. Before electrode placement, the skin was cleaned with ethanol. The location of the electrodes was based on anatomical landmarks, as previously described [34]. Cinematic and EMG data were recorded concurrently.

Each registration session was performed with the patient seated comfortably on a high backed-chair, and instructed to look steadily at a target affixed on the wall, 90 cm away from the eyes, to avoid movement of the head. The measurements were performed in a quiet and comfortable environment. Each recording began with the largest number of teeth in contact and the test bolus (soft or hard) positioned on the center of the surface of the tongue; subsequently, the patient was instructed to start chewing and, after 10 s, to stop. Each registration session comprised the following 18 different recordings: 9 with a soft bolus (3 sets of 3 recordings, as follows: 1 chewing on the right side, 1 chewing on the left side, and 1 chewing freely), and 9 with a hard bolus (3 sets of 3 recordings, as follows: 1 chewing on the right side, 1 chewing on the left side, and 1 chewing freely). The soft bolus was represented by a sugar-free chewing gum (chosen in mint or strawberry flavor by the patient), previously chewed until a uniform soft consistency was achieved; the hard bolus was represented by a wine gum of similar size (cola flavored), replaced with a new gum after each of the 9 recordings. The dimensions of the soft and hard boluses were 20 mm × 1.2 mm × 5 mm; the soft bolus weighed 2 g and the hard bolus weighed 3 g.

### 2.3. Signal Analysis

The kinematic signals were analyzed using custom-made software (University of Turin, Italy) that allows for automatic data segmentation and analysis. This approach has been described elsewhere [31]. The first cycle, during which the bolus was transferred from the tongue to the dental arches, was excluded from the analysis. Jaw movements between two consecutive masticatory pauses were also excluded, if they did not represent a chewing cycle based on the presence of at least one of the following characteristics: (i) minimum opening smaller than 4 mm; (ii) duration shorter than 300 ms; (iii) vertical opening smaller than 3 mm. From each cycle, the following variables were extracted: (i) closing angle (the angle formed by the tangent to the closing phase of the cycle and the horizontal reference line); (ii) cycle height (the distance between the apex and the inversion, between the opening and closing trace); (iii) maximum cycle width (the maximum horizontal distance between the opening and the closing trace); (iv) maximum lateral excursion (the maximum horizontal distance of the closing trace from the vertical axis); (v) cycle duration (the time needed for the trace to leave the apex and find it again) [35,36]. The values computed for each variable were averaged over all cycles, recorded for the same side of mastication and the same bolus. The chewing cycles were divided into non-reverse and reverse, based on the vectorial direction of closure. The closure angle was measured between a straight line obtained by a robust regression procedure on the last part of the curve (from 2.0 to 0.1 mm, from the closing point in the vertical direction) and the horizontal line of the side of mastication. Next, cycles with a closure angle larger than 90° were grouped in the reverse set.

The surface EMG was rectified and low-pass filtered with a 10 Hz cut-off frequency (signal envelope). During each cycle, the maximum values of the EMG envelope of both sides were computed. The percent difference between ipsilateral (the deliberate chewing side) and contralateral masseter peak EMG amplitude was computed. The percent difference between ipsilateral and contralateral masseter peak EMG amplitude was calculated as an indication of the coordination between the bilateral masseter muscles. Such normalization overcomes the known limitations of the use of the EMG amplitude, and allows data to be pooled from different subjects, and for ensemble averages to be computed. The muscle onset periods were computed by a wavelet-based method for muscle on–off detection, which provides an accuracy suitable for clinical applications, and is completely automatic, without any intervention required by the operator. Next, the occlusal pause was calculated as the time difference between the end of the EMG activity of the masseter and the beginning of the next opening phase.

### 2.4. Statistical Analysis

#### 2.4.1. Comparison between Deep Bite Patients and Controls before Orthodontic Treatment

The cinematic variables were analyzed using a three-way mixed model analysis of variance (ANOVA). The repeated measures were the side of mastication (right and left), and the bolus (soft and hard); the between-group factor was the subject group (deep bite group and control group).

The EMG variables were analyzed using a four-way mixed model ANOVA. The repeated measures were the side of mastication (right and left), the bolus (soft and hard), and the side of EMG detection (right and left); the between-group factor was the subject group (deep bite group and control group).

#### 2.4.2. Comparison between Pre-Treatment and Post-Treatment Deep Bite Patients

The cinematic variables were analyzed using a three-way mixed model ANOVA. The repeated measures were the side of mastication (right and left), and the bolus (soft and hard); the between-group factor was the subject group (before treatment and after treatment).

The EMG variables were analyzed using a four-way mixed model ANOVA. The repeated measures were the side of mastication (right and left), the bolus (soft and hard), and the side of EMG detection (right and left); the between-group factor was the subject group (before treatment and after treatment).

Significance was accepted for p-values less than 0.05.

## 3. Results

This study included 81 patients with deep bite malocclusion (11.4 ± 1.1 [yr.mo]; M = 32 and F = 49), and 14 age- and gender-matched patients without malocclusion (9.11 ± 1 [yr.mo]; M = 5 and F = 9) as the control group.

### 3.1. Comparison between Deep Bite Patients and Controls before Orthodontic Treatment

**Figure 3:**
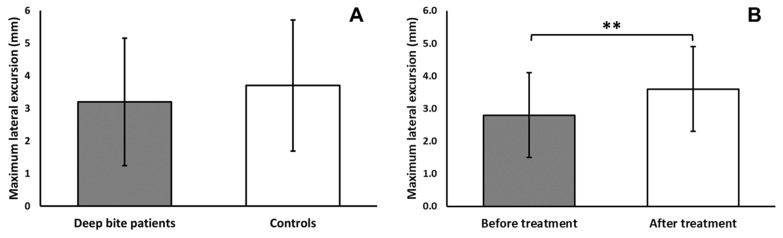
(**A**) Maximum lateral excursion in deep bite patients compared to controls, showing a tendency towards a smaller lateral component in the malocclusion group. (**B**) Maximum lateral excursion in deep bite patients, before and after treatment with FGB, showing a significant increase in the lateral component of the chewing pattern. ** *p* < 0.01.

**Figure 4:**
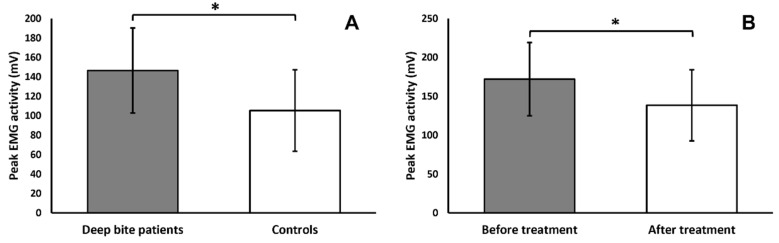
(**A**) Peak EMG amplitude of masseter and temporalis anterior (data merged) in deep bite patients compared to controls, showing significantly higher activity associated with malocclusion. (**B**) Peak EMG activity of masseter and temporalis anterior (data merged) in deep bite patients, before and after treatment with FGB, showing a significant reduction in muscular activity after correction of the malocclusion. * *p* < 0.05.

The comparison between the deep bite malocclusion group (*n* = 81) and the control group (*n* = 14), before the beginning of treatment, showed the following:

1. The variable “closing angle” was significantly dependent on bolus hardness (F = 15.55, *p* < 0.001), with smaller closing angles during hard bolus chewing.
2. The variable “cycle height” was significantly dependent on bolus hardness (F = 56.65, *p* < 0.001), with greater cycle heights during hard bolus chewing, and showed a tendency towards shorter cycles in the deep bite malocclusion group (F = 3.39, *p* < 0.069), albeit not significant.
3. The variable “maximum width” was significantly dependent on bolus hardness (F = 17.52, *p* < 0.001), with greater maximum widths during hard bolus chewing, and on the interaction between group and bolus hardness (F = 4.57, *p* < 0.05), with the deep bite malocclusion patients showing larger maximum widths during hard bolus mastication.
4. The variable “maximum lateral displacement” was significantly dependent on bolus hardness (F = 6.68, *p* < 0.05), with greater maximum lateral displacement during hard bolus chewing, and showed a tendency towards smaller maximum lateral dis-placement in the deep bite malocclusion group (F = 3.36, *p* < 0.071), albeit not significant (Figure 3).
5. The variable “duration” was significantly dependent on group (F = 3.96, *p* < 0.05), with shorter lasting cycles in the deep bite malocclusion group.
6. The variable “peak EMG amplitude” was significantly dependent on group (F = 4.20, *p* < 0.05) (Figure 4), with a higher peak EMG amplitude in the deep bite malocclusion group; on bolus hardness (F = 96.77, *p* < 0.001), with a higher peak EMG amplitude during hard bolus chewing; on the association between the side of mastication and the side of masticatory muscle (F = 82.75, *p* < 0.001); on the association between all factors (F = 8.19, *p* < 0.01).

### 3.2. Comparison between Pre-Treatment and Post-Treatment Deep Bite Patients

The comparison between the deep bite malocclusion group before treatment and the deep bite malocclusion group after correction of the malocclusion (*n* = 25) showed the following:

1. The variable “closing angle” was significantly dependent on bolus hardness (F = 20.74, *p* < 0.001), with smaller closing angles during hard bolus chewing.
2. The variable “cycle height” was significantly dependent on bolus hardness (F = 98.75, *p* < 0.001), with greater cycle heights during hard bolus chewing.
3. The variable “maximum width” was significantly dependent on bolus hardness (F = 44.54, *p* < 0.001), with greater maximum widths during hard bolus chewing.
4. The variable “maximum lateral displacement” was significantly dependent on bolus hardness (F = 11.11, *p* < 0.01), with greater maximum lateral displacement during hard bolus chewing; on group (F = 10.59, *p* < 0.01), with greater maximum lateral displacement after correction of the malocclusion (Figure 3); on mastication side (F = 5.05, *p* < 0.05), with greater maximum lateral displacement on the right side; on the association between bolus hardness and group (F = 4.50, *p* < 0.05), with greater maximum lateral displacement during hard bolus chewing, after correction of the malocclusion.
5. The variable “duration” was significantly dependent on group (F = 6.14, *p* < 0.05), with shorter lasting cycles after correction of the malocclusion.
6. The variable “peak EMG amplitude” was significantly dependent on group (F = 7.29, *p* < 0.05) (Figure 4), with a lower peak EMG amplitude after correction of the malocclusion; on bolus hardness (F = 98.55, *p* < 0.001), with a higher peak EMG amplitude during hard bolus chewing; on the association between muscle side and same-sided mastication (F = 60.53, *p* < 0.001), with a greater peak EMG amplitude in right masticatory muscles during right-sided mastication and vice versa; on the association between mastication side, bolus hardness, and group (F = 5.48, *p* < 0.05); on the association between muscle side, bolus hardness, and group (F = 6.05, *p* < 0.05).

## 4. Discussion

In this study, the chewing patterns of children, i.e., during growth, with deep bite were compared to those of children without malocclusion. Furthermore, the chewing cycles were recorded a second time, after correction of the malocclusion, in a subset of deep bite patients. To the best of our knowledge, there are no previous reports in the literature on the concomitant analysis of both the cinematics of mandibular movements during chewing, under standardized conditions, and EMG recordings of the masticatory muscles in deep bite patients.

Regarding the chewing pattern, the kinematic variables considered, related to masticatory efficiency [35] and recorded with hard and soft bolus, were dependent on bolus hardness for both the deep bite patients and controls. This means that the capability of deep bite patients to apply the load [37] does not differ from normal subjects. In other words, deep bite patients are able to adapt the chewing pattern to the bolus consistency, even though the muscular activation during chewing was significantly higher in deep bite patients compared to the control group. Moreover, the analysis of the chewing cycles indicates that the lateral component of the closing stroke is significantly constrained by the malocclusion, leading to more vertical and repetitive patterns, in accordance with earlier observations [23]. Interestingly, treatment with FGB was able to significantly increase the lateral component, indicating that correction at the occlusal level translates to a change in neuromuscular coordination.

The most evident result of this study is the hyperactivity of both the masseters and anterior temporalis muscles during chewing in growing children. This clearly shows that deep bite is not simply a dental malocclusion, but rather a cranial and muscular structure, derived from a complex mosaic of familial features. Hence, the answer to the question “Is the potential influence of the hyperactive muscles decisive for the cranial structure, or is the cranial morphology responsible for the strength of the masticatory muscles?” is complex and not univocal. There is probably an intimate interaction between the neuromuscular system and cranial development, both inherited, but deeply influenced by peripheral and individual inputs. This is the reason why, as stated in the introduction, deep bite is notoriously prone to relapse, after initially successful treatment. Even if deep bite is diagnosed as dental malocclusion, it is linked to a cranial and muscular structure that must be considered, in order to best treat these patients. Therefore, orthodontics cannot be dedicated to dental repositioning tout court, but must be aimed at controlling neuromuscular hyperactivity, especially in growing subjects.

This study has some limitations. The first concerns the small number of control subjects (*n* = 14); a perfectly physiological occlusion, or meso-occlusion, is unfrequently found in the general population, with occlusion being the result of a series of complex processes of compensation of individual characteristics, aimed at obtaining the best possible function. The second concerns the reduced number of deep bite patients who underwent recording of the masticatory function after correction of the malocclusion (*n* = 25); this was mainly the result of therapeutic choices other than FGB, and, to a lesser extent, the impossibility on the part of the patients to come back for a second recording after correction, as the participation in this study was on a completely voluntary basis.

This study showed that functional therapy with FGB is able to significantly lower the muscular hyperactivity of deep bite patients during chewing, in agreement with other studies carried out with functional removable appliances [26]. The fact that malocclusion influences neuromuscular control suggests that the orthodontic approach for deep bite should differ from that for dental crowding cases. It is important to emphasize that muscular hyperactivation may reappear and subsequently determine recurrence of the malocclusion, exposing the patient to the relative side effects. If the patient is made aware of this risk, they will be able to recognize the problem and seek treatment in time; otherwise, the recurrence may lead to significant deterioration of the teeth and of the other structures of the stomatognathic system, which is eventually irreversible in the elderly.

## 5. Conclusions

The results of this study on masticatory function during development highlight the features of deep bite malocclusion, showing that it is not only a dental malocclusion, but also affects functions well beyond the position of the teeth, and that it must be monitored during growth to prevent worsening of the stomatognathic system. In the future, this research should move to the study of the interplay between craniofacial structure and the vertical dimension, in particular, occlusion and masticatory function. 

## Data Availability

The data presented in this study are available on request from the corresponding author. The data are not publicly available due to privacy concerns.
